# Distinct Influences of Urban Villages on Urban Heat Islands: A Case Study in the Pearl River Delta, China

**DOI:** 10.3390/ijerph15081666

**Published:** 2018-08-06

**Authors:** Wei Wu, Hongyan Ren, Ming Yu, Zhen Wang

**Affiliations:** 1 College of Geographical Science, Fujian Normal University, No.8 Shangsan Road, Fuzhou 350007, China; ww20170820@gmail.com; 2 State Key Laboratory of Resources and Environmental Information System, Institute of Geographic Sciences and Natural Resources Research, Chinese Academy of Sciences, 11A Datun Road, Chaoyang District, Beijing 100101, China; wangz.16b@igsnrr.ac.cn

**Keywords:** urban village, land-use type, urban heat island, land surface temperature, Pearl River Delta

## Abstract

Widely scattered urban villages (UVs) and increasingly serious urban heat islands (UHIs) are common urban problems in highly urbanized regions, especially in the developing countries. However, the influences of UVs on UHIs remain little understood. In this study, different methodologies are performed to retrieve land surface temperature (LST) from thermal bands and the nearest object-oriented method with spectral, texture, shape metrics using ZY-3 high-resolution satellite imagery, and road network data are used to extract UVs and other land-use types in the Guangzhou–Foshan (GF) core areas of Pearl River Delta (PRD). Moreover, the relationship between LST and land-use types is then analyzed on the multiple scales. The results show that five land-use types (vegetation, normal construction land (NCL), UVs, water, and unused land) extracted by the object-oriented method were qualified for subsequent analysis because of satisfactory overall accuracy (0.887) and the Kappa coefficient (0.863). In the GF core areas presenting the most outstanding UHI effect across the PRD region, about 60.5% of the total area is covered by the impervious surfaces, including NCL (50.4%) and UVs (10.1%). The average LST of UVs was 1.89–2.97 °C lower than that of NCL. According to the average contribution index of thermal effect and the Pearson’s correlation coefficients, UVs present a relatively lower contribution to UHI and a weaker warming effect than NCL, but possess a higher contribution to UHI and a stronger warming effect than other land-use types, resulting in some slightly lower LST-valleys in the UVs adjacent to the NCL and distinct LST-peaks of UVs close to vegetation and water on the surface temperature profile lines. This work increases our understanding of the relationship between increasingly serious UHIs and widely distributed UVs, and would be valuable for local authorities to monitor and improve urban environment in metropolitan regions.

## 1. Introduction

In recent years, rapid urbanization leads to temperature rise in cities all around the world, forming an urban heat island (UHI). In addition to thermal discomfort, urbanization in the developing world often leads to the problem of informal settlements (e.g., urban villages (UVs) and slums) [1,2,3,4]. However, the relationships between these informal urban settlements and abnormal urban thermal environment remain little understood.

Dramatic transformations and reconstruction of urban space were induced by the living and production demands of ever increasing population during the rapid urbanization. A large number of rural villages on edges of cities can become engulfed, leaving the rural settlements intact and scattered throughout the urban area [2,5,6,7,8]. The development of UVs is neither authorized nor scientifically planned. As a result, UVs are commonly featured by small and dense substandard buildings, absent vegetation coverage, poor sanitary conditions, overcrowded population, and environment pollution [9,10], which attract increasing attention from local urban planners and policymakers who concentrate on the urban renewal and better living environment.

The UHIs are defined by the temperature differences between urban areas and suburban/rural areas [11,12,13,14,15]. Urbanization leads to the transformation of natural landscapes, such as vegetation, water bodies, and agrarian lands, into impervious surfaces, such as construction land. This transformation reduces vegetation evapotranspiration, increases solar radiation absorption, influences the local and regional climate, and gives rise to the UHI [16]. The UHI can be evaluated with air and surface temperatures. The studies of UHI based on air temperatures were mainly to evaluate the intensity of heat islands [17] and simulate the urban temperatures using the Weather Research and Forecasting model [18,19] or Met office Unified model [20]. However, air temperature data are obtained from meteorological stations, and their sparse distribution makes a spatially continuous analysis difficult [21]. Therefore, numerous studies of UHI have been mainly analyzed based on land surface temperature (LST), which can be derived from thermal infrared bands from Landsat [22,23,24], Moderate Resolution Imaging Spectroradiometer (MODIS) [25] or Advanced Spaceborne Thermal Emission and Reflection Radiometer (ASTER) [26].

The relationship between LST and land use/land cover (LULC) has been a recent research hotspot, which can provide a scientific basis for urban planning and the regulation of regional environment. Numerous studies have shown that the spatiotemporal patterns of UHIs were influenced to various degrees by different land-use types [22,27,28]. Among them, the construction land makes the largest contribution to the UHI effect, while forest land, farmland, parks, and water bodies tend to mitigate the UHI effect [29,30,31]. However, UVs are seldom considered as a separate category from construction land to quantitatively analyze the effect of UVs on UHIs. The main reason is that the mapping of UVs is difficult indeed because unplanned development leads to complex spectral and spatiotemporal patterns [10]. Recently, a few studies of informal settlements in developing countries have used high-resolution remotely sensed data to detect it. Hofmann et al. [8] and Rhinane et al. [32] used an object-oriented approach to detect informal settlements and the precision of the classification was satisfactory. Owen and Wong [33] proposed to detect informal settlements with spectral, texture, geomorphology and road accessibility metrics, and indicated that roads and texture play a key role in depicting the differences between informal and formal settlements.

In this study, we perform to detect UVs based on the object-oriented classification method with spectral, texture, shape metrics using ZY-3 high-resolution remotely sensed imagery and road data. Moreover, we investigate the spatiotemporal patterns of UHIs in the core areas of Guangzhou–Foshan (GF) metropolitan area in the Pearl River Delta (PRD) region, using LST data retrieved from Landsat thermal bands, and explore the contribution of UVs to the UHI effect on the multiple scales. This study would provide support for dynamically monitoring the ecological effect of UVs, and preventing some UVs-related public health risk and the regulation of regional environment.

## 2. Study Areas and Data

**Figure 1:**
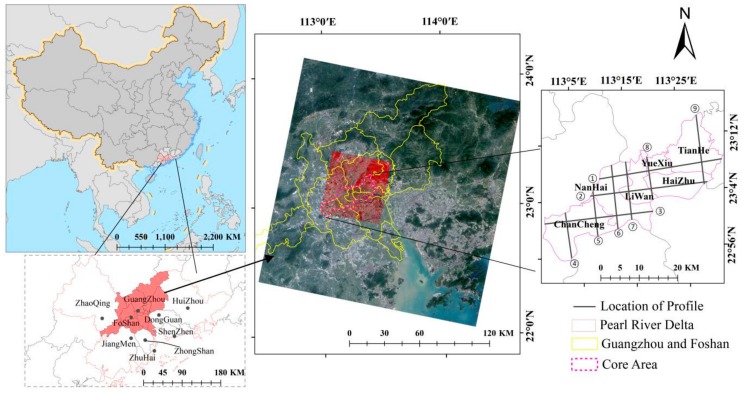
Study areas and satellite data coverage of the Guangzhou-Foshan (GF) in China and locations of profile lines. The data covered consist of the following satellite images (large to small): Landsat 8 Operational Land Imager (OLI) (RGB 321), ZY 3 (RGB 432).

The PRD, with the GF metropolitan area as the central region, located in Southern China (21°27′–23°56′ N, 111°59′–115°26′ E), covers a total area of 44,700 square kilometers (Figure 1). It has a marine subtropical monsoon climate, an annual average temperature of 20–22 °C, and an annual rainfall of 1720 mm, with rain and higher temperatures occurring during the same summer period. In 2017, the gross domestic product of the PRD region reached one trillion dollars, and the permanent resident population reached 59.98 million at the end of that year [34]. The PRD, in particular the GF region, has a large number of UVs during the rapid urbanization over the past decades. For example, Guangzhou City has 138 UVs, while Shenzhen has more than 1000, with 200 within the Special Economic Zone [35].

Table 1 outlines the data employed for this study. Before retrieving LST, radiation calibration was performed on the MODIS data, along with geometric correction and cropping of the imagery. For the Landsat 5/8 data (Appendix A), radiometric calibration and Fast Line-of-sight Atmospheric Analysis of Spectral Hypercubes (FLAASH) atmospheric correction were performed. We used ZY-3 satellite data to obtain land-use type data including that of the UVs in the core areas of GF. This data set includes multispectral and panchromatic images with spatial resolutions of 5.8 m and 2.1 m, respectively. Prior to extraction, we performed geometric corrections and orthorectifications, as well as fusion processing of multispectral and panchromatic images by Pan Sharpening. In addition, road data from Open Street Map (OSM) were used as ancillary data to extract land-use types.

## 3. Methods

**Figure 2:**
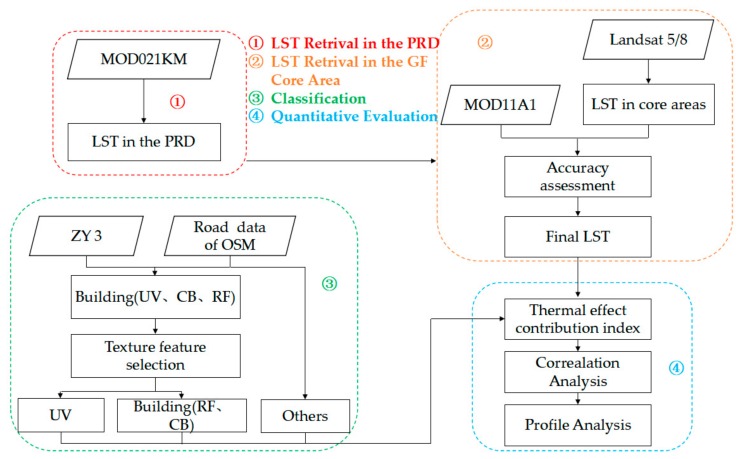
Overall flowchart of exploring the distinctive influences of UVs on urban heat islands (UHIs) (UV: urban village, RF: roofs of factories, CB: conventional buildings).

As shown in Figure 2, the data-processing procedure involved in this paper mainly includes: (1) retrieve LST based on MODIS data as well as UHI spatial pattern analysis of the PRD region; (2) retrieve LST from Landsat-5 thematic mapper (TM) and Landsat-8 thermal infrared sensor (TIRS), and UHI spatial pattern analysis in typical UHI core areas of PRD; (3) extract UVs and other land-use types in typical UHI core areas; and (4) analyze contribution difference of UVs and other land-use types to UHIs. Among them, (1) and (2) relate to retrieval of LST using remote sensing images. We first performed LST retrieval and UHI spatial pattern analysis of the PRD region using remotely sensed imagery with a spatial resolution of 1 km. We also selected a typical UHI core area of PRD (i.e., core areas of GF) and performed LST retrieval and UHI spatial pattern analysis using remote sensing images with a higher spatial resolution (30 m). Then, we extracted land-use type data from high-resolution remotely sensed imagery for the selected areas (step 3). Finally, we comprehensively analyzed the influence and contribution of each land-use type in the selected areas, especially UVs, to the regional UHI. These procedures will be further described below.

### 3.1. Land Surface Temperature Retrieval from Thermal Infrared Data

As mentioned above, this paper discusses two levels of LST retrieval.

(1) LST retrieval in the PRD region. We retrieved LST of the entire PRD region from MODIS thermal infrared bands with a resolution of 1 km and based on a split window 1 (SW1) algorithm [36]. First, we extracted the 31st, 32nd, 1st, 2nd, and 19th bands of the PRD region. Second, we employed the SW1 method to retrieve LST on 22 September 2011, 11 October 2013, 18 October 2015, and 23 October 2017, respectively. During this process, we adopted estimated brightness temperature, surface emissivity, and atmospheric transmittance. On this basis, due to the errors caused by time phase, we used the standard deviation classification method to eliminate the influence of errors on LST spatial pattern change [37]. The LST was classified into seven categories: lowest, low, sub-low, medium, sub-high, high, and highest temperatures. The zones with the highest, high, and sub-high temperatures were considered as UHI zones. Meanwhile, this study introduced the urban heat island ration index (URI) [38]. The URI was described as shown in Equation (1), where *m* is the normalization level index; *i* is the temperature level of urban areas higher than that of the suburb; *n* is the number of temperature levels of urban areas higher than that of the suburb; *w* is the weight value, which is the level of the *i*th level; and *p* is the percentage of the *i*th level.
  (1) $$URI=\frac{1}{100m}\sum_{i=1}^{n}w_{i}p_{i}$$
(2) LST retrieval of the typical UHI area. We retrieved LST of typical UHI area from Landsat thermal infrared bands with a resolution of 30 m (Landsat-5 TM and Landsat-8 TIRS), using different algorithms. First, we used the radiative transfer equation (RT), single channel method (SC) [39], and mono-window algorithm (MW) [40] to retrieve surface temperature from Landsat-5 TM data acquired on 21 September 2011; for the Landsat-8 TIRS data acquired on 18 October 2015, we added a split-window 2 (SW2) algorithm [41]. Because no actually measured data for the surface temperature were used, we adopted the data in the same period of MODIS LST products to verify the retrieval results, in order to select an appropriate retrieval algorithm for Landsat-8 data acquired on 12 October 2013 and 23 October 2017.

### 3.2. Extraction of Land-Use Types

According to the spatiotemporal pattern of UHI within the PRD region, a typical UHI area is to be selected for extracting land-use type information. The proposed method includes three parts, which are described successively. All analyses were conducted using eCognition Developer 8.7 (Trimble, Trappentreustr, München, Germany).

#### 3.2.1. Multi-Scale Segmentation of Images Combined with OSM Road Data

**Figure 3:**
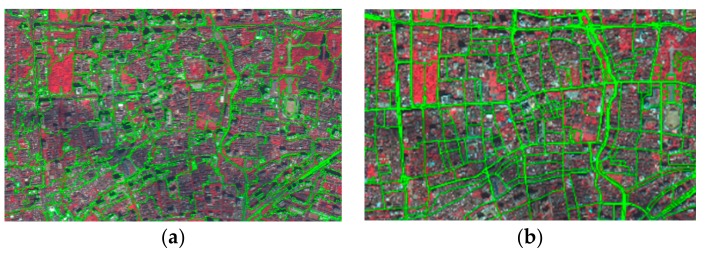
Comparison of segmentation results. (**a**) Results without Open Street Map (OSM) road data; (**b**) results with OSM road data.

Multi-scale segmentation is a bottom-up method that combines adjacent pixels or small-segmented objects to ensure the minimum average heterogeneity exists between the objects and maximum homogeneity exists between intra-object pixels, in order to achieve image segmentation based on local fusion. The segmentation scale range was set to be 290. Considering the high concentration of buildings in UVs, the shape factor was set to 0.5, and the smoothness and compactness weights were 0.5. UVs appeared spatially as blocks divided by road networks. In this study, we added adaptive auxiliary data (e.g., OSM road data) in the segmentation process to make it possible to highlight the distinguishing features of the ground objects, while retaining the auxiliary data’s adaptability to the spatial characteristics of UVs (Figure 3).

#### 3.2.2. Extraction of Land-Use Types Based on the Nearest Neighbor Method

Before adopting the nearest neighbor classification method, it is necessary to establish the spectral, spatial, and textural rules of each land-use type.

First, we considered both the spectral and shape features. Among the nine features initially selected, seven were spectral features and inversion indicators, including four multispectral bands (blue, green, red, near-infrared indicators), brightness, maximum difference scaling and normalized difference vegetation index (NDVI); two were object shape features, which were the shape index and length/width. Based on the classification sample and classification system, we optimized these features. Based on the optimal feature dimensions, we calculated the degree of separation of each feature dimension for different object types, and performed a preliminary classification, where we divided the land-use types into buildings (e.g., UVs, conventional buildings (CBs), and roofs of factories (RFs)), road, unused land, vegetation, and water.

**Figure 4:**
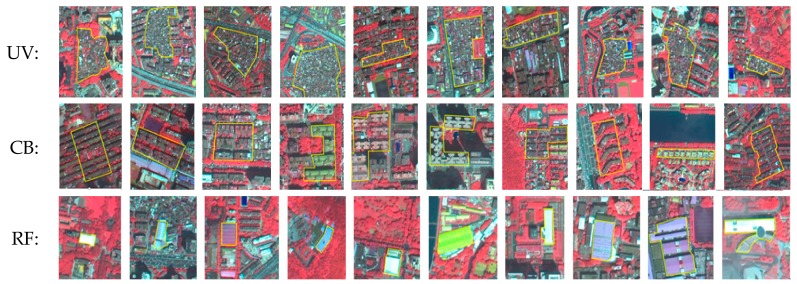
Sample selection: UV, CB and RF (From top to bottom).

In addition, we used the gray-level co-occurrence matrix (GLCM) method to extract the texture features of UVs [42,43] to supplement the spectral and shape features to extract UVs from buildings. The GLCM method describes precise textures by analyzing the spatial correlation of gray scales with high computational efficiency. Therefore, based on the preliminary classification of buildings, we collected 30 samples, ten each for UVs, CBs, and RFs (Figure 4).

To improve the efficiency of UV extraction, we performed *t*-test to compare the differences in eight textures in UVs, CBs, and RFs (Table 2). From the *t*-test results, we can see that the contrast, dissimilarity, StdDev, and mean textures of UV were not significantly different from those of other objects. Therefore, we excluded these four textures. We selected the remaining four textures as alternatives: homogeneity 90°, angular second moment (Ang. 2nd moment) 45°, entropy 90°, and correlation 90°.

Finally, based on the feature space optimization tool of eCognition Developer 8.7, we selected four textures, four spectral bands, and two feature shapes to optimize the feature selection. According to the feature differentiation index, we obtained the optimal feature dimensions and further divided the buildings into UVs, CBs, and RFs.

#### 3.2.3. Accuracy Verification

This paper selected 531 verification sample points by referring to Google Earth imagery and the random point selection methods, and used the Training and Test Area (TTA) Mask file and confusion matrix analysis methods of eCognition Developer 8.7 to evaluate the extraction accuracy of land-use type data. The kappa coefficient and producer’s accuracy/user’s accuracy were computed for the assessment. The producer’s/user’s accuracy is the ratio of the number of correctly classified samples to the number of reference/classification samples. The kappa coefficient was written as shown in Equation (2):

(2) $$Kappa=\frac{N\sum_{i=1}^{r}x_{ii}-\sum_{i=1}^{r}\left(x_{i+}\times x_{+i}\right)}{N^{2}-\sum_{i=1}^{r}\left(x_{i+}\times x_{+i}\right)}$$

where *r* is the number of rows in the confusion matrix of classification; $x_{ii}$ is the number of along the diagonal; $x_{i+}$ is the total number of row *i*; $x_{+i}$ is the total number of column *i*; and *N* is the total number of cells.

### 3.3. Evaluation of Contributions of Different Land-Use Types to Heat Island Effects

This study aims to use the contribution index of thermal effect to quantitatively evaluate the contribution of different land-use types to the heat island effect in the core urban area of GF region. The contribution index of thermal effect ($H_{i}$) refers to the degree of impact that the surface temperatures of different land-use types have on the region’s average temperature, i.e., the contribution of each land-use type to the region’s heat island effect. $H_{i}$ and the contribution of the initial heat island effect ($H_{i}^{\prime }$) were shown in Equations (3) and (4):

(3) $$H_{i}=\frac{H_{i}^{\prime }}{\sum_{i=1}^{n}H_{i}^{\prime }}*100\%$$

(4) $$H_{i}^{\prime }=\frac{\sum_{j=1}^{n}\left(T_{ij}-T_{a0}\right)*n_{i}}{T_{a0}N}$$

where $T_{ij}$ is the temperature of the *j*th pixel with temperatures above the average regional temperature in the land category *i*; $T_{a0}$ represents the average temperature of the region during imaging; $n_{i}$ is the number of pixels in the land category *i*, of which the temperature is higher than the average regional temperature; and *N* represents the region’s land area. To simplify the comparison, we performed data normalization and obtained the contribution indices of different land-use types.

In addition, in order to further study the relationships of surface temperature to UVs, normal construction land, water, and vegetation, this paper randomly selected 40 points inside the UVs and generated buffers with radii of 0.2, 0.5, 0.8, and 1 km. We analyzed the relationship between the area proportion of different land-use types and surface temperature in each buffer zone, using Pearson’s correlation coefficients.

### 3.4. Profile Analysis of Land Surface Temperature in Typical Urban Heat Island Areas

The temperature profile analysis can be used to explore the internal structure of the heat islands in downtown and urbanized areas. However, it can also be used for analyzing the differences in LST between urban and rural lands, as well as the effect of urban expansion on heat islands [44]. Using ArcGIS 10.3 (ESRI, Redlands, CA, USA) software, this study randomly selected nine surface temperature profile lines from west to east and from north to south (locations of profile lines and serial numbers of profile lines in Figure 1). Based on the ZY-3 high-resolution remotely sensed imagery, we subdivided the land-use types on each profile line and marked them with different colors, so that we could more intuitively and accurately analyze the differences in surface temperature profile lines between different land-use types.

## 4. Results

### 4.1. Spatiotemporal Patterns of Urban Heat Island

**Figure 5:**
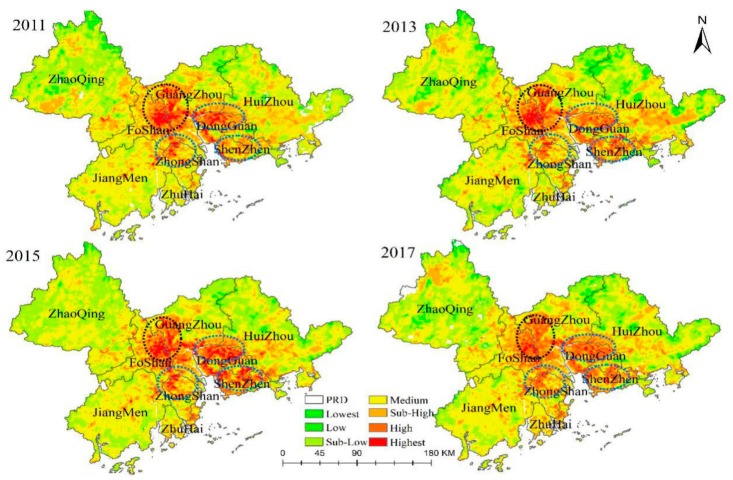
Spatiotemporal patterns of land surface temperature (LST) in the Pearl River Delta (PRD) region with four typical areas on 22 September 2011, 11 October 2013, 18 October 2015 and 23 October 2017.

The LST exhibited clear spatial differentiation characteristics in the PRD region. As illustrated in Figure 5, the zones with the highest, high, and sub-high temperatures (i.e., UHI zones) were mainly distributed in four areas, the GF core areas, the Guangzhou–Dongguan city interface, Shenzhen, and the Zhongshan–Zhuhai city interface. Meanwhile, the lowest and low temperature zones were mainly distributed in Zhaoqing, Northern Huizhou, and Northern Guangzhou. The spatial pattern of LST in the PRD region has not changed significantly during 2011–2017. Accordingly, the core areas of GF were considered as a typical area for further analysis of UHI in detail.

**Figure 6:**
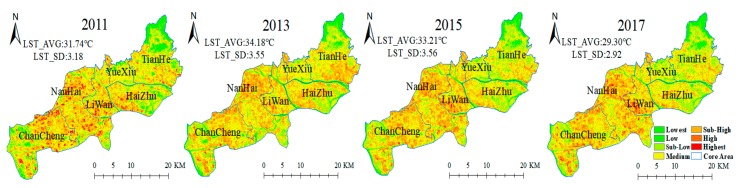
Spatiotemporal patterns of LST in the core urban areas of GF on 22 September 2011, 11 October 2013, 18 October 2015 and 23 October 2017. (LST_AVG: average LST; LST_SD: LST standard deviation).

The comparisons of the LST retrieval results using different algorithms and the MODIS LST products were shown in the Supplementary Files (the scatter plots in Figures S1 and S2 and statistic parameters in Table S1). Similarly, UHI zones in the GF core areas were also spatially differentiated. As shown in Figure 6, these zones were observed in each administrative district, like Liwan, Nanhai and Changchneg Districts. In particular, UHI zones in 2015 and 2017 were spatially clustered near the interface of Liwan and Nanhai Districts. In contrast, the zones with lower temperature, rather than those with the sub-low temperature level, were mainly distributed in the northern part of Tianhe District, the eastern part of Haizhu District, and the western part of Chancheng District. Meanwhile, the UHI effect presented an overall downward trend according to the URI decreasing to 0.2031, as well as the slightly decline of the UHI area proportion from 32.06 to 30.34% (Table 3). These results showed that the UHI effect in the GF core areas was mitigated to some degree in the past 7 years.

### 4.2. Results and Analysis of Land-Use Types

According to the overall accuracy (0.876) and the Kappa coefficient (0.851) (Table 4), the land-use types were well extracted from the ZY-3 high-resolution remote sensing images. As far as the producer’s and user’s accuracy were concerned, the UVs possessed slightly lower accuracies (77.6% and 84.6%) than those of vegetation (94.7% and 98.2%), water (98.6% and 97.2%), and roads (89.0% and 87.2%), although the omission and commission of UVs have been appropriately controlled by the texture selection procedure. In a word, the present extraction accuracy can meet the demands of subsequent analysis. Then, the roads, CBs, and RFs were combined into the normal construction land (NCL) type, resulting in the five major land-use types including NCL, UVs, unused land, vegetation, and water.

**Figure 7:**
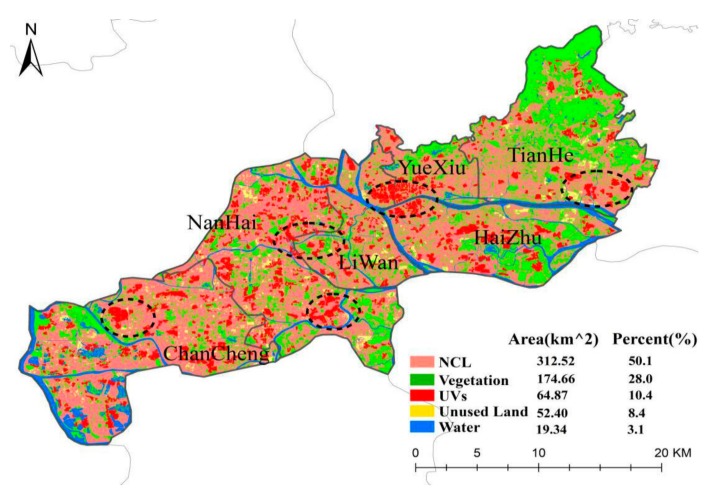
Classification result in the core urban areas of GF.

In terms of urban space, the GF core areas were highly urbanized. According to the area ratio of five land-use types as illustrated in Figure 7, more than 60% of the core areas were covered by impervious surfaces including NCL (50.1%) and UVs (10.4%). In comparison, about 31% of this region was covered by permeable surfaces (vegetation, 28.0%; water area, 8.4%). Similar to the spatial patterns of UHI zones, UVs also appeared in each administrative district. There were several zones with spatially clustered UVs as labeled by black dotted ovals in Figure 7, including the zones near the Pearl River fork between Yuexiu and Haizhu Districts, the Wangjiegang forest park in Chancheng District, the Pingzhou Branch of Provincial People’s Hospital in Nanhai District. These results indicated that the GF core areas were featured by widely distributed UVs.

### 4.3. Contributions of Different Land-Use Types to Urban Heat Island

The average surface temperature and UHI contribution index ($H_{i}$) varied with the land-use types across the GF core areas. The five land-use types were ranked from the highest to the lowest surface temperature values as follows (Table 5): NCL > unused land > UVs > vegetation > water, which persisted during 2011–2017. The average surface temperature of NCL was 1.89–2.97 °C higher than that of UVs. In comparison, the sequence of $H_{i}$ for these land-use types was slightly different: NCL > UVs > vegetation > unused land > water, and this order also remained unchanged in this period. These results showed that different land-use types made different contributions to the spatial patterns of UHI across the GF core areas, and NCL contributed the most to the UHI effect across the GF core urban areas, followed by UVs.

In addition, the average regional LST was correlated with the proportions of area of various land-use types. According to the Pearson’s correlation coefficients in Table 6, the average LST values decreased significantly when the proportion of area covered by the permeable surfaces (vegetation and water) increased in the UV-centered buffer zones. Meanwhile, surface temperature increased remarkably when those of area covered by the impervious surfaces (NCL and UVs) were higher in these buffer zones. In contrast to the significantly positive influence of NCL on the surface temperature, the contribution of UVs was not remarkable, which means that UVs and NCL possessed weak and strong warming effects in the core areas, respectively.

Lastly, the influences of land-use types on the spatial patterns of UHI could be further understood according to the analysis of the profile lines across the GF core areas.

**Figure 8:**
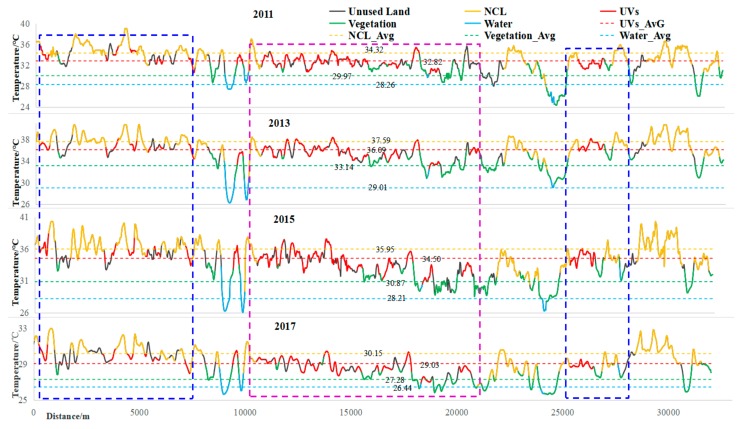
Profile analysis results of Line 1 (NCL_AVG: average surface temperature of normal construction land; UVs_AVG: average surface temperature of UVs; Vegetation_AVG: average surface temperature of vegetation; Water AVG: average surface temperature of water).

On the LST profile line 1 (see the other profile lines in the Supplementary Files in Figure S3), the surface temperature values for all 5 land-use types formed a waving curve from 2011 to 2017 (Figure 8). The average temperature of all profile lines in 2011–2017 were ranked from the highest to the lowest as follows: NCL > UVs > Vegetation > Water. As the largest contributor to the UHI effect, NCL was mainly indicated by outstanding peaks on the profile lines. Water and vegetation appeared as obvious valley-shapes due to their relatively low surface temperature. UVs tended to be featured by clear valley shapes when they were adjacent to NCL (labeled by blue dotted rectangles) on the profile lines. However, when adjacent to the vegetation and water bodies (labeled by purple dotted rectangles), UVs showed a small peak shape on the profile lines, indicating an UHI phenomenon. In the GF core areas, the areas with small peak shapes were, for example, near the Zhujiang fork between Yuexiu and Haizhu Districts, nearby the Guangjun Business Center in Haizhu District, and near Huolushan Park in Tianhe Park. The results indicated that UVs were widespread and mainly surrounded by NCL, so that the slight but clear surface temperature valleys could be presented in this highly urbanized region.

## 5. Discussion

To our knowledge, our study concentrated on the effects of widely scattered UVs on increasingly serious UHIs in some metropolitan regions for the first time. In this study, we analyzed the influences of UVs, as well as other land-use types, on the spatiotemporal patterns of UHI in the GF core areas by means of multi-source remote sensing data. This study will help to understand the spatiotemporal patterns of UHI in the urban area with numerous UVs, which can provide local environmental and hygienic authorities with useful clues about environmental enhancement and related public health management.

It has been proved that the object-oriented classification method is an effective solution for detecting the informal settlements (e.g., UVs and slums) [8,32], as well as the scenarios-based classification [10], landscape metrics and transfer learning [9]. However, the development of UVs in China has seldom been reported or analyzed using the object-oriented classification method. In addition, image segmentation is still difficult and challenging [45,46,47]. Numerous studies indicated that image segmentation efficiency was improved when adding some auxiliary data, such as digital elevation models, cadastral, population, or road network [48,49]. In our study, OSM road data was employed as auxiliary data for improving the efficiency of ZY-3 high-resolution remotely sensed imagery segmentation. Moreover, the extraction of texture using the GLCM method was used to enrich the features of ground objects to overcome the disadvantages of shape and spectral features in identifying UVs from CBs and RFs. As a result, UVs and other land-use types were effectively extracted in the GF core areas with an overall accuracy of 0.876 and the Kappa coefficient of 0.851. The results indicated that the nearest object-oriented method had strong adaptability and high efficiency and could be considered as an alternative approach to detect UVs in China.

The UHI is an outstanding environment problem due to its profound influence on the lives of urban residents, and some research have focused on how to mitigate it [50,51,52,53]. In our study, the UHI effect in the GF core areas was mitigated to some degree in the past 7 years. It is due to the fact that the joint GF development that has taken place in recent years has been accompanied by continuous implementation of policies protecting and restoring green spaces and water areas for the improvement of the ecological structure of the GF core areas. In addition, previous studies have pointed out that the construction land is a predominant contributor and an important influencing factor of UHIs, while vegetation and water can mitigate heat islands in the urban area [22,27,28]. These findings are similar to our study that NCL presented the highest ground temperature, as well as the largest contribution to UHIs in the GF core areas. In comparison, UVs, as a specific land-use type separated from the construction land, possessed 1.89–2.97 °C lower surface temperature and a relatively weaker warming effect, which may be related to the buildings’ tile roofs of UVs with higher reflectance than cement roofs or their unique roofs with many potted plants [54]. In addition, the slight but clear surface temperature valleys could be presented from surface temperature profile analysis results. These results implied that the specific urban settlement (i.e., UVs) should be considered as a separate land-use type when the influences of LULC on UHIs were to be investigated. It would supply useful reference for monitoring and managing urban environment.

In fact, UVs are widely distributed in not only the GF core areas but also the other cities (e.g., Shenzhen City) in the highly urbanized PRD region. In addition to the relatively lower temperature, UVs are commonly featured by poor sanitation, overcrowding population, low coverage public health services [9,10], resulting in a high environmental suitability for some biological vectors (e.g., *Aedes Albopictus*), as well as the vector-borne diseases in these urban regions [55,56]. Accordingly, UVs should be heavily emphasized due to their serious environmental health risk. Together with the quick detection of UVs by using remote sensing, the unique influences of UVs on UHIs investigated in this study would provide meaningful clues for monitoring biological vectors, as well as preventing and controlling UVs-related infection in these urban areas with numerous UVs.

A few limitations of this study warrant mention. First, much earlier UVs information before 2012 should be obtained for the future analysis of the spatiotemporal variations of UVs, although the urban reconstruction focusing on the demolishing UVs was slowed down after the 2010 Asian Games. Additionally, the extraction accuracy of all the land-use types could be further improved through adding many more auxiliary data (i.e., openly accessible point of interest (POI) data). Finally, ground temperature at night may be spatially different from that of daytime across the urban area and the analysis of their difference could be conducted in future research to provide more meaningful support for monitoring and regulating urban environment.

## 6. Conclusions

In summary, this paper draws the following conclusions:

(1) This study, for the first time, systematically verified the distinctive effect of the spatial distribution of UVs on UHI, which was mainly characterized by the lower surface temperature and relatively weaker warming effect of UVs than those of NCL.
(2) Multi-scale segmentation combined with road data and the extraction of UVs based on the nearest neighbor method with spectral, shape and texture metrics provided methodological reference for the detection of informal settlement (e.g., slums or UVs) in future research.
(3) This study provides theoretical support for the dynamic monitoring, transformation, and redevelopment of UVs, preventing some UVs-related public health risk as well as the regulation of the regional environment.

## Figures and Tables

**Table 1:** Overview of the multitemporal data.

Data	Date	Resolution	Source	Purpose
MOD021KM MOD11A1	22 September 2011	1 km	https://ladsweb.modaps.eosdis.nasa.gov/search/	To retrieve LST in the PRD region and verify the accuracy of LST in the GF core areas
	11 October 2013			
	18 October 2015			
	23 October 2017			
Landsat-5 or 8	21 September 2011	30 m	http://www.gscloud.cn/	To retrieve more detailed LST in the GF core areas
	12 October 2013			
	18 October 2015			
	23 October 2017			
ZY 3	14 April 2014	5.8 m/2.1 m	http://www.cresda.com/CN/	To extract UVs and other land-use types in the GF core areas
Road data from Open Street Map	/	/	http://www.openstreetmap.org	To serve as auxiliary data for extracting land use information

**Table 2:** Student’s *t*-test of texture feature differences.

Relationship	Homogeneity	Contrast	Dissimilarity	Entropy	Ang. 2nd Moment	Mean	StdDev	Correlation
UV–CB_all	1.078	−0.881	−1.291	2.731 *	−0.538	0.686	−1.514	−0.822
UV–RF_all	−1.92	0.019	0.900	2.465 *	−0.611	0.384	−1.251	−1.408
CB–RF_all	−2.657 *	0.869	1.855	−0.306	−0.145	−0.330	0.544	−0.333
UV–CB_0°	0.859	−1.463	−1.911	2.685 *	−2.206	0.585	−1.791	0.833
UV–RF_0°	−1.722	−0.502	0.203	2.275 *	−1.795	0.602	−1.273	0.251
CB–RF_0°	−2.765 *	0.937	1.605	−0.516	0.556	−0.004	0.711	−0.28
UV–CB_45°	1.607	−1.146	−1.576	2.752 *	−2.286 *	0.739	−1.527	−0.159
UV–RF_45°	−1.389	−0.626	0.055	2.327 *	−1.874	0.461	−1.717	−0.776
CB–RF_45°	−2.197	0.663	1.258	−0.562	0.744	−0.256	0.432	−0.327
UV–CB_90°	−0.296	1.208	0.868	2.854 *	−2.198	0.460	−1.009	−3.400 **
UV–RF_90°	−2.330 *	1.474	2.198	2.470 *	−2.06	−0.298	−0.432	−2.489 *
CB–RF_90°	−2.405 *	0.037	1.864	−0.544	0.754	−0.913	0.384	0.802
UV–CB_135°	2.008	−0.826	−1.369	2.741 *	−2.286 *	0.803	−1.468	−1.341
UV–RF_135°	−1.193	0.540	0.954	2.269 *	−1.801	0.469	−1.452	−1.74
CB–RF_135°	−1.805	1.122	2.071	−0.526	0.617	−0.390	0.569	−0.868

* This value is significant at the level of 0.05. ** This value is significant at the level of 0.01.

**Table 3:** Variation of graded area of LST in the core areas of GF.

Class	2011		2013		2015		2017	
	Area/km^2^	Per/%	Area/km^2^	Per/%	Area/km^2^	Per/%	Area/km^2^	Per/%
Lowest	6.525	1.05	5.36	0.86	1.26	0.20	2.60	0.42
Low	34.21	5.48	45.77	7.33	46.57	7.46	38.01	6.09
Sub-low	127.51	20.43	106.77	17.11	121.64	19.49	129.97	20.83
Medium	255.79	40.99	261.12	41.84	255.47	40.94	264.12	42.32
Sub-high	176.71	28.32	176.07	28.21	162.47	26.04	149.22	23.91
High	22.03	3.53	27.66	4.43	33.68	5.40	35.48	5.68
Highest	1.30	0.21	1.31	0.21	2.95	0.47	4.70	0.75
URI	0.2346		0.2416		0.2370		0.2031	

**Table 4:** Confusion matrix of land classification.

Class	Vegetation	Road	Water	Unused Land	UV	CB	RF	Sum
Vegetation	54	0	0	0	1	0	0	55
Road	1	89	0	0	5	4	3	102
Water	0	0	143	2	2	0	0	147
Unused land	1	4	2	42	4	8	1	62
UV	0	5	0	2	66	5	0	78
CB	1	2	0	1	2	23	0	28
RF	0	0	0	1	5	4	48	58
Sum	57	100	145	48	85	44	52	531
*Producer’s accuracy*	0.947	0.890	0.986	0.875	0.776	0.523	0.923	
*User’s accuracy*	0.982	0.872	0.972	0.677	0.846	0.821	0.827	
Overall accuracy	0.876							
Kappa	0.851							

**Table 5:** Average surface temperatures and thermal effect contribution indices of thermal effect for different land-use types (AVG: average; SD: standard deviation).

Class	2011		2013		2015		2017	
	AVG ± SD	$H_{i}/\%$	AVG ± SD	$H_{i}/\%$	AVG ± SD	$H_{i}/\%$	AVG± SD	$H_{i}/\%$
Vegetation	29.42 ± 2.61	3.00	32.57 ± 2.93	2.90	31.21 ± 2.76	1.91	27.78 ± 2.41	1.30
Water	28.33 ± 1.82	0.01	28.82 ± 2.84	0.01	28.06 ± 2.86	0.01	25.97 ± 1.84	0.01
NCL	34.88 ± 2.66	92.85	38.71 ± 2.60	92.67	36.80 ± 2.91	93.57	32.59 ± 2.47	95.03
UVs	32.99 ± 1.72	3.78	35.74 ± 1.74	4.08	34.74 ± 1.81	4.17	30.31 ± 1.50	3.29
Unused Land	33.08 ± 2.71	0.36	35.56 ± 2.60	0.34	35.75 ± 2.37	0.34	30.40 ± 2.02	0.37

**Table 6:** Pearson’s correlation coefficients of surface temperature and area ration of different land-use types.

Year	Impervious Surfaces	NCL	UVs	Permeable Surfaces
2011	0.604 **	0.292 **	0.194	−0.680 **
2013	0.943 **	0.220 **	0.189 *	−0.694 **
2015	0.525 **	0.182 *	0.147	−0.591 **
2017	0.427 **	0.230 **	0.060	−0.500 **

* This value is significant at the level of 0.05. ** This value is significant at the level of 0.01. NCL and UVs were classified into the category of impervious surfaces while the permeable surfaces included water and vegetation.

## Supplementary Materials

The following are available online at http://www.mdpi.com/1660-4601/15/8/1666/s1. Figure S1: Scatter plot of different methodologies to retrieve surface temperature from Landsat-5 TM and MODIS LST product on 21 September 2011, Figure S2: Scatter plot of different methodologies to retrieve surface temperature from Landsat-8 TIRS and MODIS LST product on 18 October 2015, Figure S3: Temperature profile analysis results of Line 2–9, Table S1: Different methodologies to retrieve surface temperature from Landsat-5 TM and Landsat-8 TIRS.

## Appendix A

List of Landsat-5/8 images used for LST retrieval according to their path, row, date (dd/mm/yyyy) and scene name.

**Table A1:** List of Landsat images used in this study.

Path	Row	Date	Landsat Scene
122	44	21/09/2011	LT51220442011264BKT00
122	44	12/10/2013	LC81220442013285LGN01
122	44	18/10/2015	LC81220442015291LGN00
122	44	23/10/2017	LC81220442017296LGN00

**Table A2:** List of acronyms.

Acronyms	Acronyms of Full Name	Acronyms	Acronyms of Full Name
ASTER	Advanced Spaceborne Thermal Emission and Reflection Radiometer	OSM	Open Street Map
AVG	Average	PRD	Peral River Delta
CB	Conventional buildings	RF	Roofs of factories
FLAASH	Fast Line-of-sight Atmospheric Analysis of Spectral Hypercubes	RT	Radiative transfer equation
GF	Guangzhou and Foshan	SC	Single channel method
GLCM	Gray level co-occurrence matrix	SD	Standard deviation
LST	Land surface temperature	SW	Split window
LULC	Land use/Land cover	TIRS	Thermal Infrared Sensor
MW	Mono window	TM	Thematic mapper
MODIS	Moderate resolution Imaging Spectroradiometer	TTA	Training and Test Area
NCL	Normal construction land	UHI	Urban heat island
NDVI	Normalized difference vegetation index	URI	Urban ratio index
OLI	Operational Land Imager	UVs	Urban villages
