# Supplementary material for: Distinct Influences of Urban Villages on Urban Heat Islands: A Case Study in the Pearl River Delta, China
Source: Int J Environ Res Public Health. 2018 Aug 6;15(8):1666. doi: 10.3390/ijerph15081666 (PMC6121422; doi:10.3390/ijerph15081666)
Supplement: Supplementary file 1 [file ijerph-15-01666-s001.pdf]

In this paper, the statistical results of the minimum, maximum, average and standard deviation values are used to evaluate the retrieval LST results. Then, we randomly selected 500 validation sample points and made a scatter plot of different methodology to retrieve surface temperature from Landsat-5/8 data and MODIS LST product. According to the comparison of the LST retrieval results using different algorithms and the MODIS LST products (the scatter plots in Figure S1, S2 and statistic parameters in Table S1), the SC-algorithm was found to be more appropriate for the retrieval of LST from Landsat-5 TM while the LST retrieval based on the SW2-algorithm produced better results for Landsat-8 TIRS data. For this reason, SC-algorithm to retrieve LST from Landsat-5 TM in 2011 and SW2-algorithm to retrieve LST from Landsat 8 TIRS in 2013, 2015, and 2017 was used in the following analysis.

Table S1. Different methodology to retrieve surface temperature from Landsat-5 TM and Landsat-8 TIRS (radiative transfer equation (RT), single channel method (SC), mono-window algorithm(MW), split Window2 (SW2))

Retrieval algorithm	Min	Max	Mean	SD
**Landsat 5 in September 21, 2011**				
RT	22.39	40.61	30.16	3.55
MW	16.75	44.20	22.09	3.64
SC	21.74	39.86	28.99	3.49
MODIS data	22.73	37.19	29.32	2.45
**Landsat 8 in October 18, 2015**				
RT	23.34	51.30	29.01	3.07
MW	22.83	50.21	28.58	3.12
SC	21.47	42.90	25.81	2.36
SW2	24.25	40.26	30.96	3.48
MODIS data	25.69	38.91	31.73	2.47

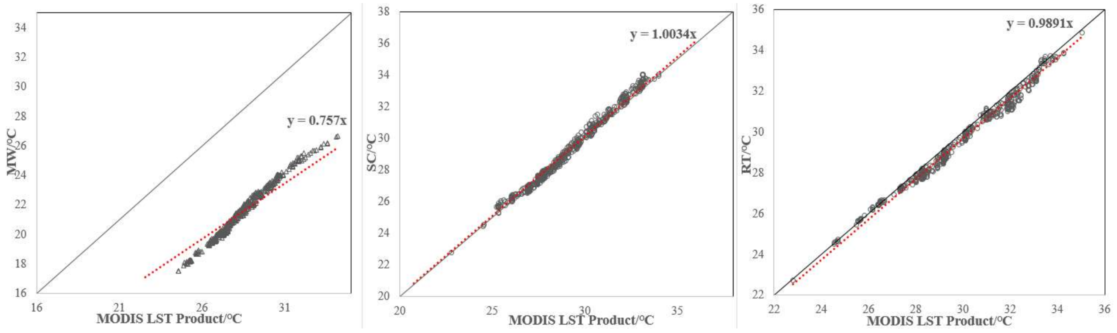

(**a**) (**b**) (**c**)

Figure S1. Scatter plot of different methodology to retrieve surface temperature from Landsat-5 TM and MODIS LST product in September 21, 2011: (**a**) mono-window algorithm; (**b**) single channel method; (**c**) radiative transfer equation.

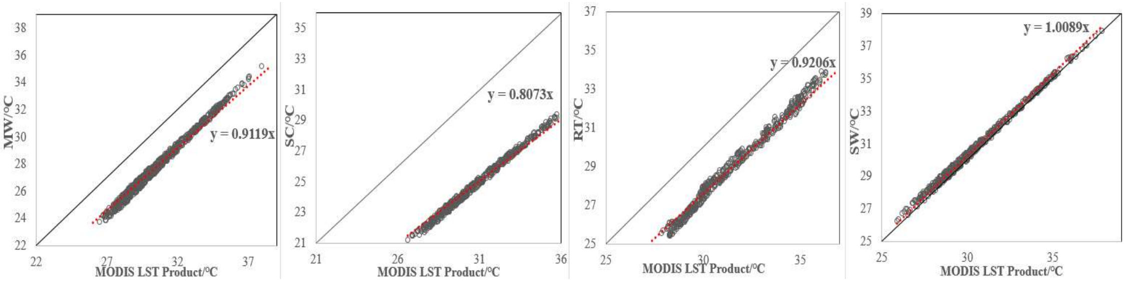

(**a**) (**b**) (**c**) (**d**)

Figure S2. Scatter plot of different methodology to retrieve surface temperature from Landsat-8 TIRS and MODIS LST product in October 18, 2015: (**a**) mono-window algorithm; (**b**) single channel method; (**c**) radiative transfer equation; (**d**) split window algorithm.

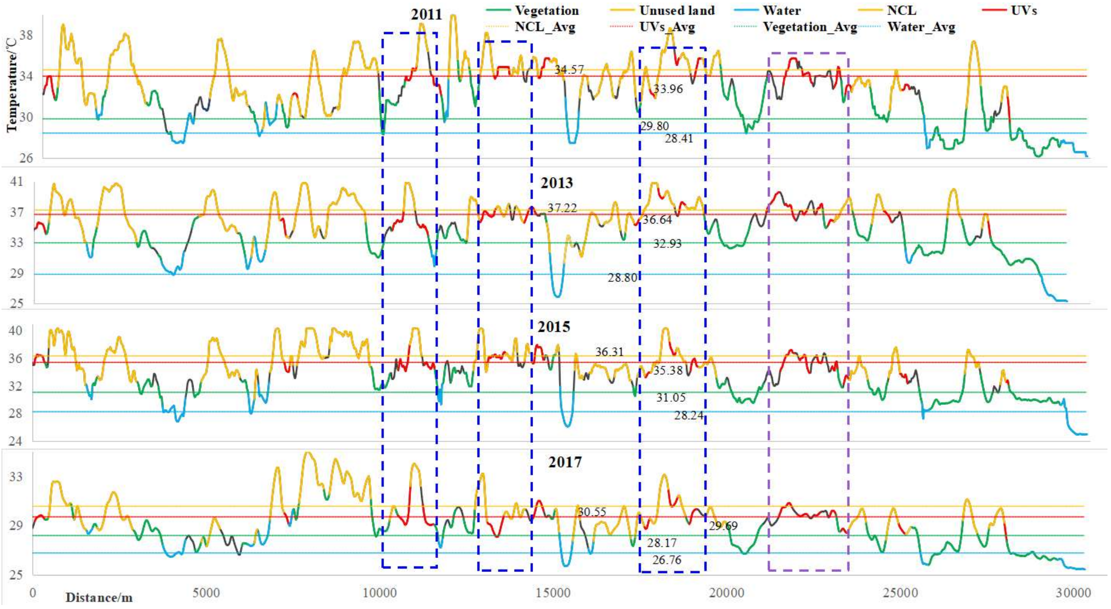

(**a**)

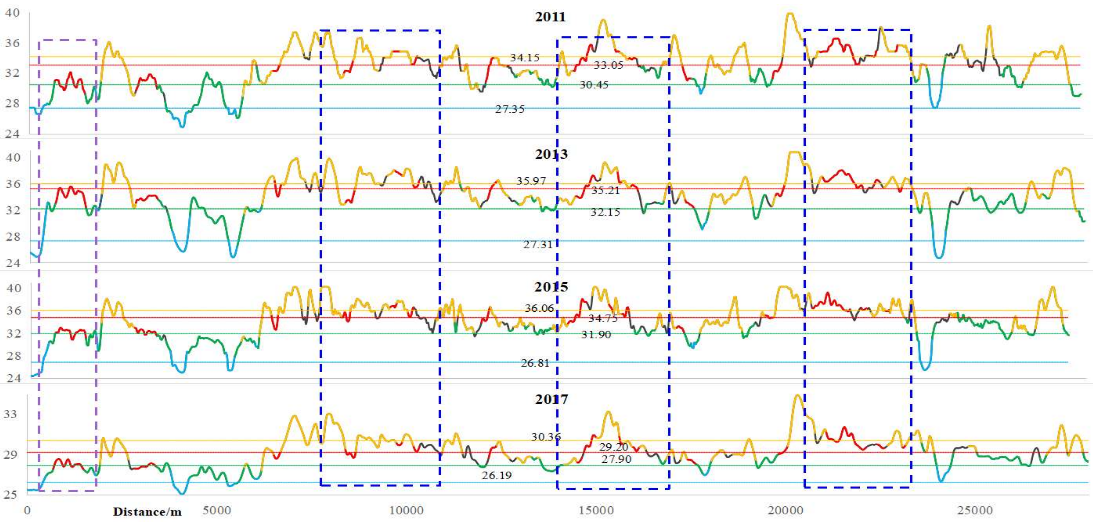
2011
34.15
33.05
30.45
27.35
2013
35.97
35.21
32.15
27.31
2015
36.06
34.75
31.90
26.81
2017
30.36
29.20
27.90
26.19
Distance/m

(**b**)

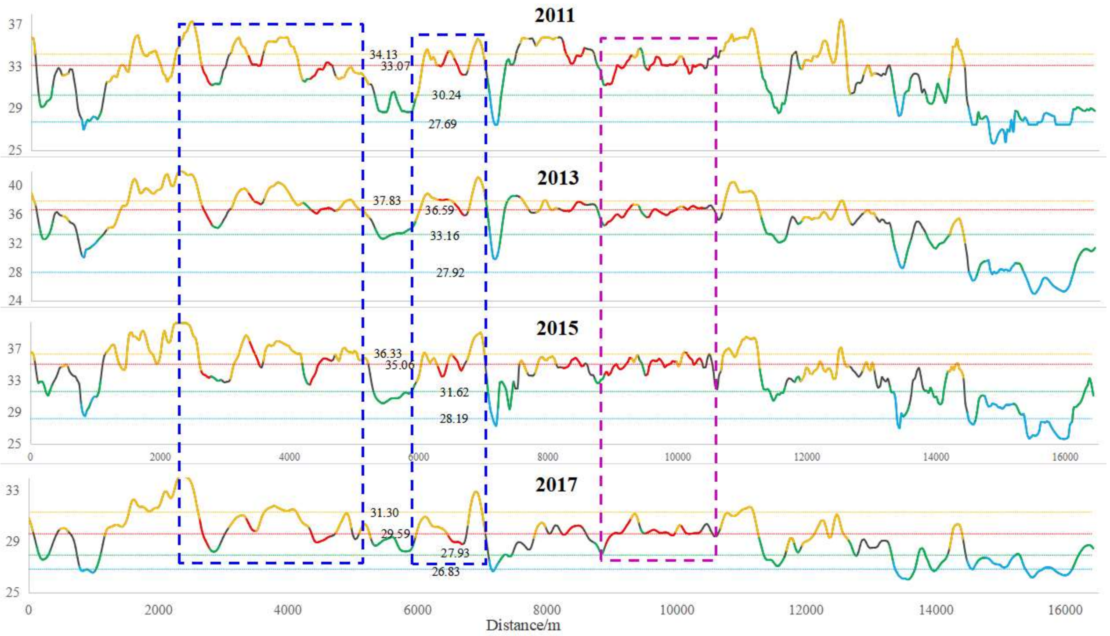
2011
34.13
33.07
30.24
27.69
2013
37.83
36.59
33.16
27.92
2015
36.33
35.06
31.62
28.19
2017
31.30
29.59
27.93
26.83
Distance/m

(**c**)

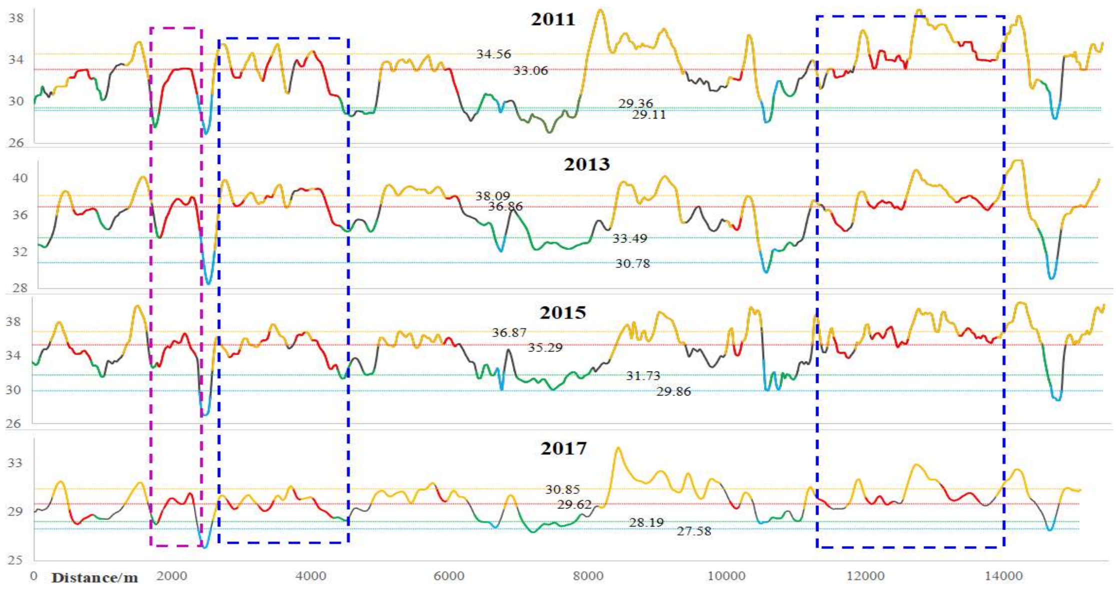
2011
34.56
33.06
29.36
29.11
2013
38.09
36.86
33.49
30.78
2015
36.87
35.29
31.73
29.86
2017
30.85
29.62
28.19
27.58
Distance/m
0
2000
4000
6000
8000
10000
12000
14000

(**d**)

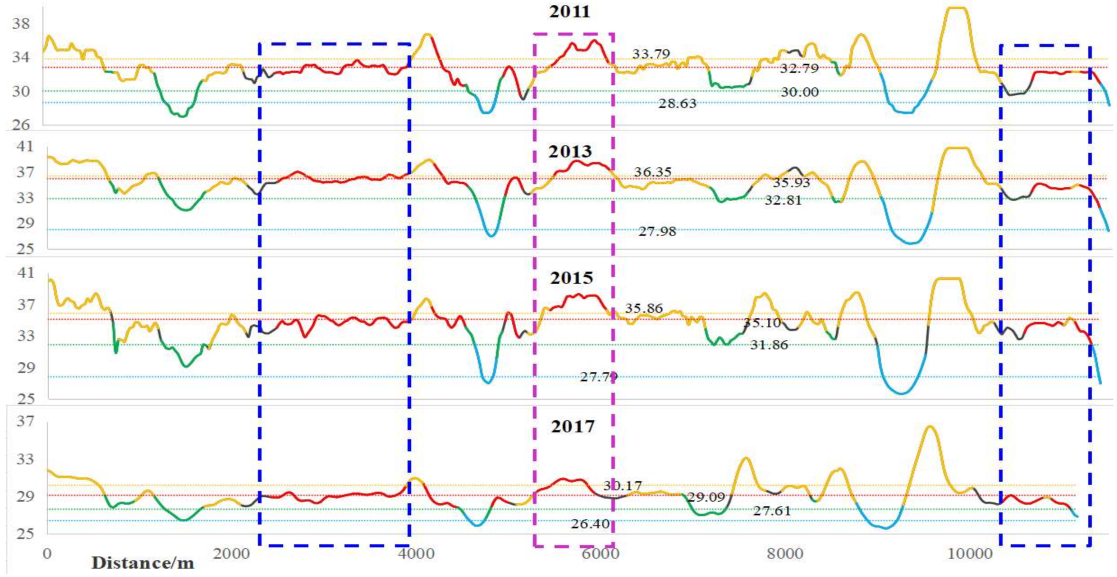
2011
33.79
32.79
30.00
28.63
2013
36.35
35.93
32.81
27.98
2015
35.86
35.10
31.86
27.79
2017
30.17
29.09
27.61
26.40
Distance/m
0
2000
4000
6000
8000
10000

(**e**)

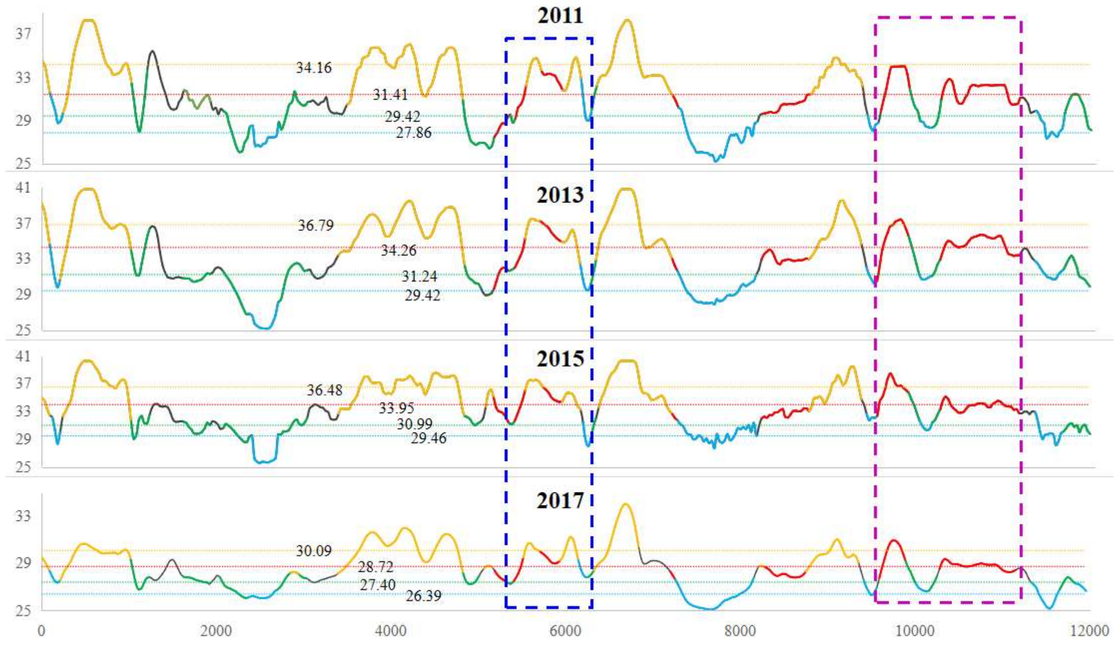
2011
34.16
31.41
29.42
27.86
2013
36.79
34.26
31.24
29.42
2015
36.48
33.95
30.99
29.46
2017
30.09
28.72
27.40
26.39
0
2000
4000
6000
8000
10000
12000

(**f**)

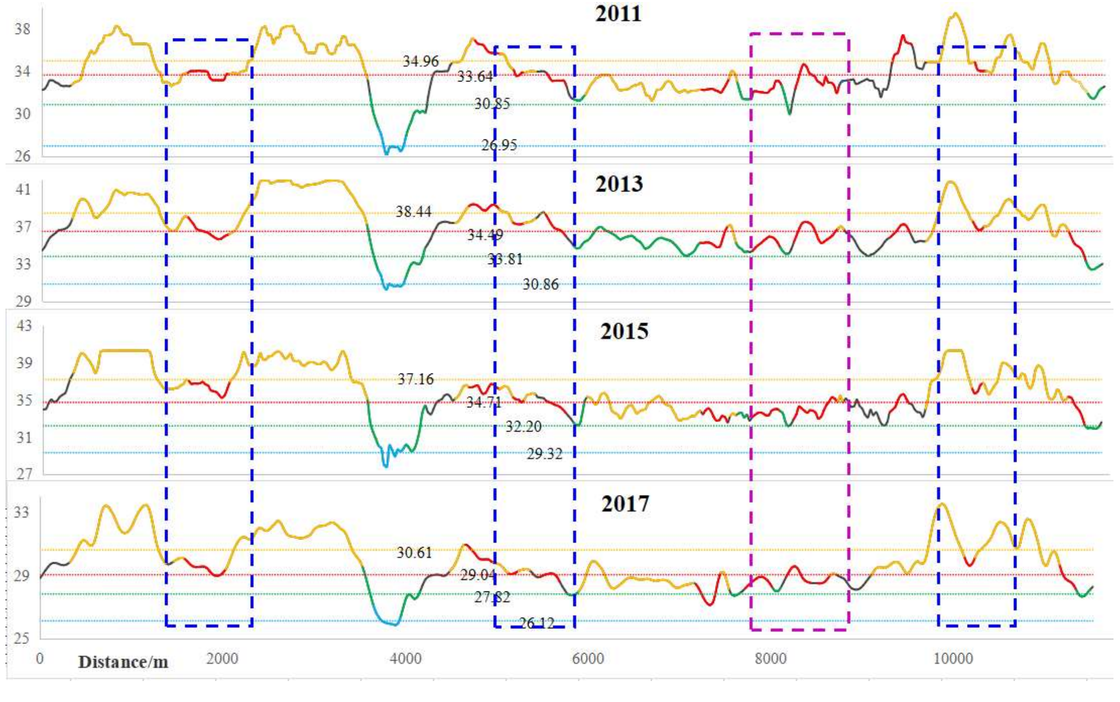
2011
34.96
33.64
30.85
26.95
2013
38.44
34.49
33.81
30.86
2015
37.16
34.71
32.20
29.32
2017
30.61
29.04
27.82
26.12
Distance/m
0
2000
4000
6000
8000
10000

(**g**)

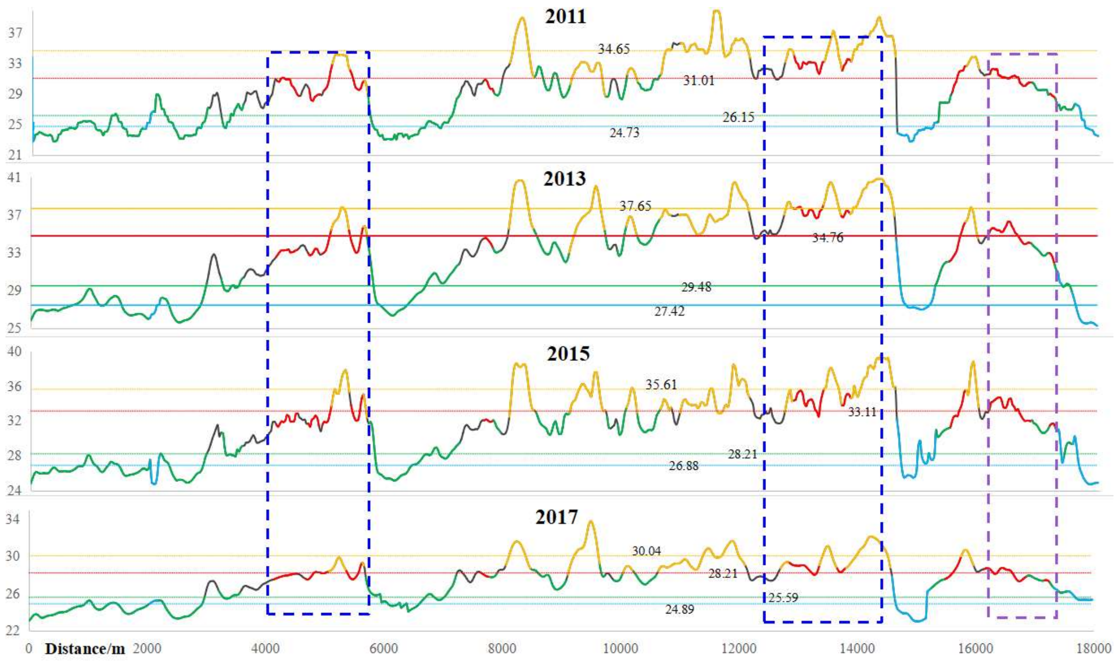

(**h**)

Figure S3. Temperature Profile analysis results of Line 2-9:(**a**) Line 2; (**b**) Line 3; (**c**) Line 4; (**d**) Line 5; (**e**) Line 6; (**f**) Line 7; (**g**) Line 8; (**h**) Line 9 (NCL_AVG : average LST of normal construction land; UVs_AVG: average LST of UVs; Vegetation_AVG: average LST of vegetation; Water_AVG: average LST of water).
